# Serum metabolic profiles in overweight and obese women with and without metabolic syndrome

**DOI:** 10.1186/1758-5996-6-40

**Published:** 2014-03-20

**Authors:** Petri K Wiklund, Satu Pekkala, Reija Autio, Eveliina Munukka, Leiting Xu, Juha Saltevo, ShuMei Cheng, Urho M Kujala, Markku Alen, Sulin Cheng

**Affiliations:** 1 Department of Health Sciences, University of Jyväskylä, Jyväskylä FIN-40014, Finland; 2 Department of Medical Rehabilitation, Oulu University Hospital, Oulu, Finland; 3 Department of Signal Processing, Tampere University of Technology, Tampere, Finland; 4 Ningbo University School of Medicine, Ningbo, China; 5 Central Hospital Central Finland, Jyväskylä, Finland; 6 Institute of Health Sciences, University of Oulu, Oulu, Finland

**Keywords:** Obesity, Metabolic syndrome, Metabolomics, Women

## Abstract

**Objective:**

To identify serum biomarkers through metabolomics approach that distinguishes physically inactive overweight/obese women with metabolic syndrome from those who are metabolically healthy, independent of body weight and fat mass.

**Methods:**

We applied nuclear magnetic resonance spectroscopy-based profiling of fasting serum samples to examine the metabolic differences between 78 previously physically inactive, body weight and fat mass matched overweight/obese premenopausal women with and without MetS. MetS was defined as the presence of at least three of the following five criteria: waist circumference ≥88 cm, serum triacylglycerol ≥1.7 mmol/L, and high density lipoprotein cholesterol (HDL-C) <1.30 mmol/L, blood pressure ≥ 130/85 mmHg and fasting glucose ≥5.6 mmol/L). Principal component analysis was used to reduce the large number of correlated variables to fewer uncorrelated factors.

**Results:**

Two metabolic factors were associated with MetS independent of BMI, fat mass, waist circumference and physical activity/fitness. Factor comprising branched-chain amino acids (BCAA) and aromatic amino acids (AAA) and orosomucoid was associated with all clinical risk factors (p < 0.01 for all).

**Conclusion:**

Two metabolic factors distinguish overweight/obese women with metabolic syndrome from those who are metabolically healthy independent of body weight, fat mass and physical activity/fitness. In particular, factor comprising BCAA, AAA and orosomucoid seems auspicious biomarker determining metabolic health as it was associated with all clinical risk factors. Further research is needed to determine the public health and clinical significance of these results in terms of screening to identify those at greatest cardio-metabolic risk for whom appropriate intervention strategies should be developed.

## Background

Excess fat mass is often seen in conjunction with a constellation of other cardiovascular risk factors such as hypertension, dyslipidemia and hyperglycemia, so-called metabolic syndrome (MetS) [1]. In recent years the prevalence of MetS has increased directly with the epidemic of obesity [2]. Comparisons of obese and lean subjects have evoked several hypotheses to explain the pathophysiological pathways of obesity associated metabolic disorders including insulin resistance, systemic low-grade inflammation [3], abdominal and ectopic fat accumulation [4], and intestinal microbiota composition [5]. Experimental evidence show that dysfunctional adipose tissue have an unfavorable effect on metabolism and thereby seem to underlie some of the obesity associated metabolic morbidities such as insulin resistance and type 2 diabetes [6]. Furthermore, nutritional factors [7], poor aerobic fitness [8] and physical inactivity [9] may also contribute to the development of MetS.

However, not all obese people develop metabolic disorders. In fact, preliminary evidence suggests that 16% of the Finnish obese women [10] and ~ 20% of the general obese population [11,12] are free from metabolic disorders. Discovery of specific biomarkers in the blood associated with MetS may reveal etiological pathways and help to identify obese individuals at risk for disease. In this study, we applied nuclear magnetic resonance (NMR) spectroscopy to analyze circulating metabolites to identify biomarkers that distinguish individuals who are metabolically healthy from individuals with MetS, independent of fat mass and physical activity/fitness.

## Materials and methods

### Study subjects

One hundred and three participants were recruited from the city of Jyväskylä and its surroundings to participate in EWI-study (Exercise and weight control intervention to study aerobic exercise intervention for improving physical fitness and weight control in overweight and obese women, ISRCTN87529813). A study physician examined the physical condition of the subjects and ensured that they met the inclusion criteria: 25–50 year old premenopausal woman with a body mass index between 25 and 40 kg/m^2^, with a history of physically inactive lifestyle (participating in regular exercise ≤ 2 times/wk and ≤ 45 min/time), and without diagnosed musculoskeletal, hypertensive or cardiovascular conditions or type I/II diabetes and without any medication affecting glucose or lipid metabolism. The study protocol was approved by the ethics committee of Central Finland Health Care District. An informed consent was obtained from all subjects prior to the assessments.

From those subjects who fulfilled the basic inclusion criteria we identified individuals who had MetS defined as the presence of at least three of the following five criteria [13]: waist circumference ≥88 cm, fasting serum triacylglycerol ≥1.7 mmol/L, high density lipoprotein cholesterol (HDL-C) <1.30 mmol/L, glucose ≥5.6 mmol/L) and resting blood pressure ≥ 130/85 mmHg. Women who had none of the above (except waist circumference ≥88 cm) were categorized as metabolically healthy overweight/obese (MHO). Thirty-six out of 103 overweigth/obese women were characterized as MetS and forty-two as MHO. Twenty-five had one of the above (in addition to waist circumference ≥88 cm) and were discarded from the analysis.

### Background information

Background information including medical history and current health status was collected via self-administered questionnaires. Food consumption and intakes of total energy and energy-yielding nutrients were assessed from three day food records and analyzed using Micro-Nutrica software developed by the Social Insurance Institution of Finland and updated with data for new foodstuffs by the study nutritionist [14]. Leisure time physical activity (LTPA) of hours/week (participating in exercise such as walking, jogging, running, gym fitness, ball games, swimming, etc.) and physical inactivity hours per day (PIA, including lying down and sitting time) were evaluated using a validated self-administrated physical activity questionnaire described previously [15].

### Fitness test

Maximum oxygen uptake (VO2max, ml/kg/min) was assessed by bicycle ergometer. During tests, heart rates were assessed using ECG and respiratory gases and ventilation was measured using respiratory gas analyzer VIASYS (Healthcare Inc. USA). A specialist physician was responsible for monitoring ECG and blood pressure responses during the test and recording subject’s signs and symptoms throughout the test.

### Respiratory gas exchange analysis

The REE (kcal/day) was assessed by respiratory gas exchange analysis (GEA) using a ventilated-hood system (VIASYS Healthcare, Yorba Linda, CA, USA). Calibration of the GEA was carried out before each measurement according to the manufacturer’s instructions. The subjects were instructed to avoid any strenuous physical activity and large, energy and protein rich meals for 24 h before the laboratory visit. The subjects arrived at the laboratory in the morning after an overnight fast. After relaxing in a measurement bed for 30 min, a ventilated hood was placed over their heads. Their oxygen consumption and carbon dioxide production were measured for 20 min at 1 min intervals, in a supine position and in a thermoneutral (22–24°C) environment. The first 5 min of the data were discarded as artefacts. The REE was calculated using the modified Weir equation [16].

### Anthropometrical and body composition assessments

Body height (cm) was measured by using standardized protocols (a wall-fixed measuring device). Body weight (kg) and fat mass (FM, kg) were assessed using bio-impedance (Inbody 720, Biospace Co. ltd Seoul, South Korea). Precision of the repeated measurements expressed as coefficient of variation was, on average, 0.6% for FM. Body mass index (BMI) was calculated as weight/height^2^ (kg · m^−2^). Blood pressure (BP) was measured twice by manual oscillometric methods in the morning after sitting for 10 minutes after the subjects arrived at the laboratory. Standing waist circumference was measured twice with a tape measure and the mean value was used.

### Blood samples

Venous blood samples for biochemical analyses were taken in standardized fasting conditions in the mornings between 7 am and 9 am. Serum samples were stored frozen at -80°C until analyzed. Serum glucose, total cholesterol, HDL, triacylglycerol, alanine amino transferase (S-ALAT); aspartate amino transferase (S-ASAT) and gamma glutamyltransferase (GGT) were analyzed using the KONELAB 20XTi analyzer (Thermo Fischer Scientific inc. Waltham, MA, USA). Insulin was determined by immunofluorescence using the IMMULITE Analyser (Diagnostic Products Corporation, Los Angeles). The homeostasis model assessment of insulin resistance (HOMA-IR) index was calculated as (fasting insulin concentration × fasting glucose concentration)/22.5. The inter- and intra-assay CVs were 2.0% and 3.7% for glucose and 11% and 3.4% for insulin, respectively.

### Serum metabolomics

All serum samples were analysed using a high-throughput serum NMR metabolomics platform; the experimental protocols including sample preparation and NMR spectroscopy have been described in detail elsewhere [17,18]. This methodology has recently been applied in various large-scale epidemiological and genetic studies [19,20]. The NMR metabolomics methodology provides comprehensive quantitative information on various amino acids, glycolysis intermediates, fatty acid composition and degree of saturation and lipoprotein subclass distributions.

### Data analyses

All data were checked for normality using the Shapiro-Wilk’s W-test (PASW Statistics 18). If data were not normally distributed, the natural logarithms were used. Clinical characteristics and serum metabolites were compared using an independent-samples t-test. To ensure that the significant differences in metabolite levels between the groups was not confounded by age, waist circumference or BMI, analysis of covariance (ANCOVA) was used adjusting for the above-mentioned variables. Metabolites were denoted significant if the p-value was below 0.0005 to account for multiple testing of 100 independent tests. All assayed metabolites are shown in Additional file 1: Table S1 and Additional file 2: Table S2.

**Figure 1:**
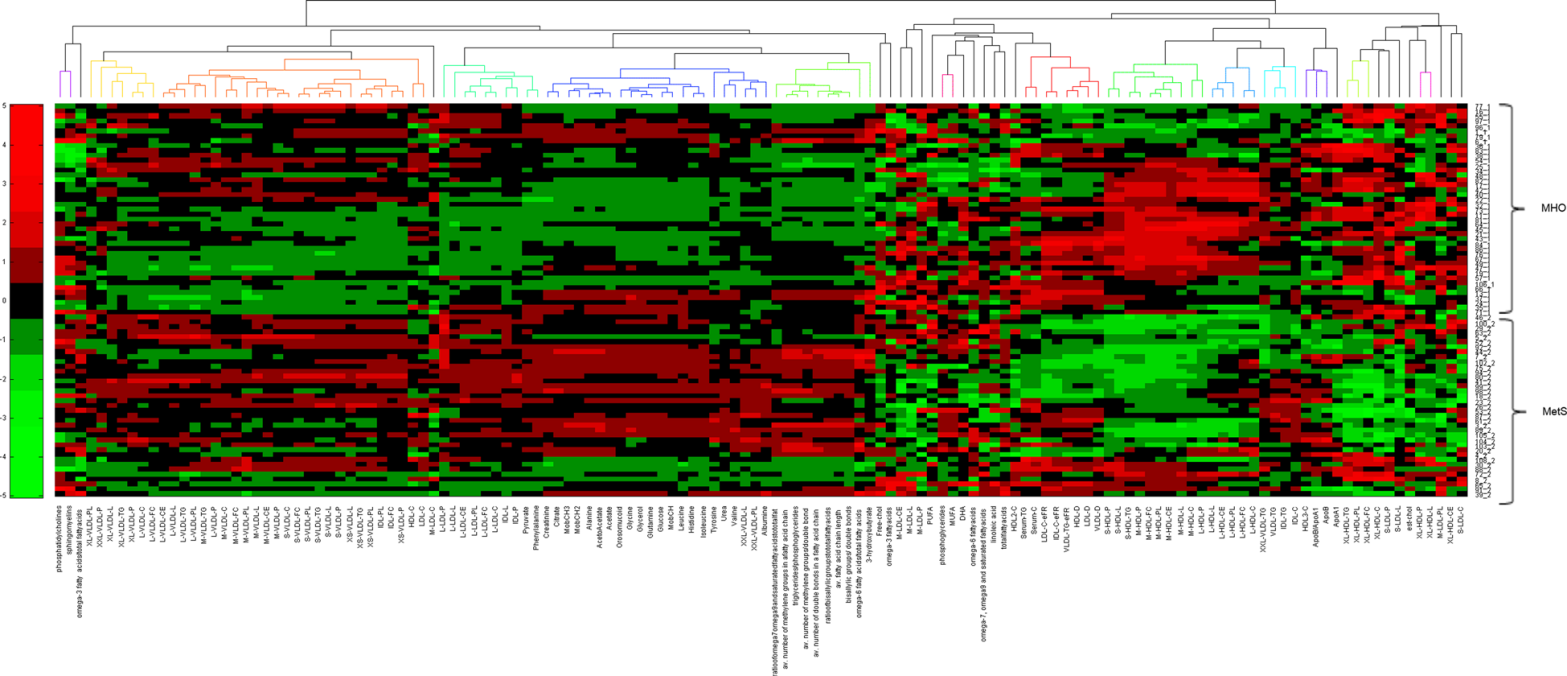
**Hierarchical clustering of metabolomics data values in MHO and MetS groups.** The heat map shows changes of x-fold standard deviation from the overall mean concentration of the metabolite in each individual belonging to either MHO or MetS group. Green squares represent a decrease, and red squares an increase. Metabolite names are shown on x-axis and individual subjects with adherent groups on (right) y-axis.

The metabolomics data was clustered utilizing hierarchical clustering algorithm. First, the metabolite and other values were metabolite-wise standardized to have 0 as a mean and 1 as standard deviation. Second, the missing values within the data were imputed with k-nearest neighbour algorithm (k = 3). The resulted data values were clustered using correlation distance and average linkage methods (Figure 1).

Given the expected multicollinearity of metabolites, we used principal component analysis (PCA) to reduce the large number of correlated variables into fewer uncorrelated factors. PCA was performed on fasting levels of amino acids, fatty acids, phospholipids, glycoproteins, ketone bodies, and glycolysis and gluconeogenesis intermediates. Varimax rotated factors with an eigenvalue ≥ 1 were identified and metabolites with a factor load ≥ 0.4 were reported as composing a given factor. Metabolomic factor scores were calculated for each individual based on the constructed scoring coefficients. Mean metabolite factor levels were compared between MHO and MetS groups with and without adjusting for age, BMI and waist circumference. Further, we assessed whether factor levels were predictors for MetS using logistic regression models in all subjects adjusted for age, waist circumference and BMI. Finally, the networks between the metabolite factors and clinical risk factors were computed with the Spearman correlation and illustrated using Himmeli software [21]. Nominal statistical significance was defined as p < 0.05.

## Results

### Clinical characteristics

The general characteristics of the study subjects are given in Table 1. MetS group were older (p < 0.005), and had higher BMI (p = 0.018) but no significant differences in other anthropometric measures, REE, VO2max, LTPA or dietary intakes of total energy and energy-yielding nutrients between the groups were found. Systolic and diastolic blood pressure, glucose, insulin, HOMA-IR, triacylglycerol, HDL, total cholesterol, S-ASAT and S-ALAT were all higher in MetS compared to MHO (p < 0.05 for all). After controlling for age, BMI and waist circumference, the statistical significance remained for all.

**Table 1:** General characteristics of the study population

	** MHO n = 42**		** MetS n = 36**		**p-value**
**Anthropometry**					
Age (years)	39.7	(7.6)	44.1	(6.1)	0.005
Height (cm)	165.5	(5.8)	164.7	(6.4)	0.565
Weight (kg)	79.1	(10.3)	83.1	(10.5)	0.095
BMI (weight (kg)/height (m)2)	28.9	(3.2)	30.6	(3.4)	0.018
Fat mass (kg)	29.0	(7.9)	32.2	(8.1)	0.071
Fat free mass (kg)	50.2	(5.5)	50.8	(5.5)	0.596
Waist circumference (cm)	95.7	(9.2)	99.2	(6.5)	0.061
**Metabolic**					
SBP (mmHg)	122.0	(7.4)	136.4	(11.3)	<0.0001
DBP (mmHg)	77.7	(6.1)	84.4	(6.7)	<0.0001
GLUC (mmol/l)	5.1	(0.3)	5.5	(0.7)	0.0001
Insulin (μIU/l)	6.4	(2.9)	9.5	(3.6)	0.0001
HOMA-IR	1.6	(1.0)	2.3	(0.9)	0.002
HDL-C (mmol/l)	1.6	(0.3)	1.4	(0.3)	0.001
TRIGLY (mmol/l)	1.0	(0.3)	2.0	(0.9)	<0.0001
CHOLtot (mmol/l)	4.7	(0.6)	5.6	(0.9)	<0.0001
ALAT (IU/l)	13.3	(5.4)	18.8	(8.9)	0.003
ASAT (IU/l)	16.5	(5.6)	19.3	(6.8)	0.038
GGT (IU/l)	22.3	(13.8)	31.5	(13.6)	0.098
**Energy expenditure and physical fitness**					
RMR (kcal/day)	1547	(200)	1505	(113)	0.337
VO2max (ml/kg/min)	31.7	(4.8)	31.3	(5.6)	0.758
LTPA (≤ ½ h/wk, %)	16		11		0.250
LTPA (1 h/wk, %)	62		46		0.671
LTPA (2 h/wk, %)	22		43		0.399
PIA (h/day)	16.1	(2.9)	16.1	(3.5)	0.965
**Diet**					
Energy (kcal)	1791	(526)	1811	(555)	0.896
Protein (E%)	18.7	(3.9)	19.1	(3.9)	0.704
Carbohydrate (E%)	44.1	(11.2)	44.5	(8.7)	0.906
Fat (E%)	33.0	(6.4)	34.5	(11.4)	0.551
Saturated fat (E%)	12.4	(3.3)	13.0	(3.1)	0.537
Monounsaturated fat (E%)	11.1	(2.5)	11.2	(4.8)	0.914
Polyunsaturated fat (E%)	6.0	(2.2)	7.1	(4.7)	0.317

Data are given as mean (SD). P-values are for 2-tailed t-tests. MHO = healthy overweight/obese; MetS = metabolic syndrome; SBP = systolic blood pressure; DBP = diastolic blood pressure; HOMA-IR = homeostatic model assessment of insulin resistance; S-ALAT = alanine amino transferase; S-ASAT = aspartate amino transferase; S-GGT = gamma glutamyltransferase; RMR = resting metabolic rate, VO2max (maximum oxygen uptake); LTPA = leisure time physical activity, PIA = physical inactivity, E% = percentage of total energy intake.

### Serum metabolites

A cluster analysis of serum metabolites implicated accumulation of several fatty acid species, VLDL lipoprotein subclasses, and glycoprotein and branched-chain amino acids in subjects with MetS (Figure 1). All metabolite and lipoprotein subclass quantities and statistics are shown in Additional file 1: Table S1 and Additional file 2: Table S2.

To further identify relevant biomarkers associated with Mets, we used principal component analysis. Eight metabolic factors were identified composed of correlated metabolites (Additional file 3: Table S3). Mean metabolite component levels are shown in Table 2. There were significant differences between MHO and MetS for factor 1 (branched-chain amino acids, phenylalanine, tyrosine and orosomucoid) (p = 0.001) and factor 2 (total fatty acids, omega-6 fatty acids, omega-7 and omega-9 fatty acids, linoleic acid, mono-unsaturated fatty acids, total phosphoglycerides, total phosphocholines) (p = 0.003). After adjusting for age, waist circumference and BMI, the level of statistical significance remained for both factors.

**Table 2:** Mean metabolite factor levels in MHO and MetS groups

**Factor**	**MHO n = 42**		**MetS n = 36**		**p-value**	**adj p-value**
1) Amino acids and glycoproteins	−0.34	(0.98)	0.43	(0.86)	0.001	0.001
2) Fatty acids and phospholipids	−0.31	(0.82)	0.39	(1.08)	0.003	0.002
3) PUFA	−0.16	(1.06)	0.21	(0.89)	0.120	0.631
4) Ketone bodies	0.07	(1.05)	−0.09	(0.94)	0.497	0.377
5) Gluconeogenic intermediates	0.06	(0.97)	−0.08	(1.04)	0.555	0.944
6) Miscellaneous	0.04	(0.97)	−0.05	(1.05)	0.726	0.464
7) Miscellaneous	0.08	(0.99)	−0.10	(1.01)	0.431	0.700
8) Miscellaneous	0.15	(0.75)	−0.18	(1.23)	0.167	0.228

MHO = metabolically healthy overweight/obese; MetS = metabolic syndrome. Values are given as mean (SD); Factor 1(leucine, isoleucine, valine, tyrosine, phenylalanine, orosomucoid); Factor 2 (total fatty acids, omega-6 fatty acids, omega7 and 9 fatty acids, linoleic acid, mono-unsaturated fatty acids, total phosphoglycerides, total phosphocholines); Factor 3 (docosahexaenoic acid, polyunsaturated fatty acids, omega-3 fatty acids); Factor 4 (acetoacetate, 3-hydroxybutyrate); Factor 5 (glutamine, glycine, pyruvate); Factor 6 (acetate, histidine); Factor 7 (creatinine, citrate); Factor 8 (urea). P-values are for the difference in mean metabolite factor levels between MHO and MetS groups with and without adjustment for age, fat mass and waist circumference.

To verify that the association between metabolite factors with MetS was not confounded by differences in age and body fat, we performed a logistic regression analysis adjusted for age, waist circumference and BMI with MetS as the dependent (outcome) variable. The results showed that both factors were significantly associated with MetS (p < 0.01 for both).

**Figure 2:**
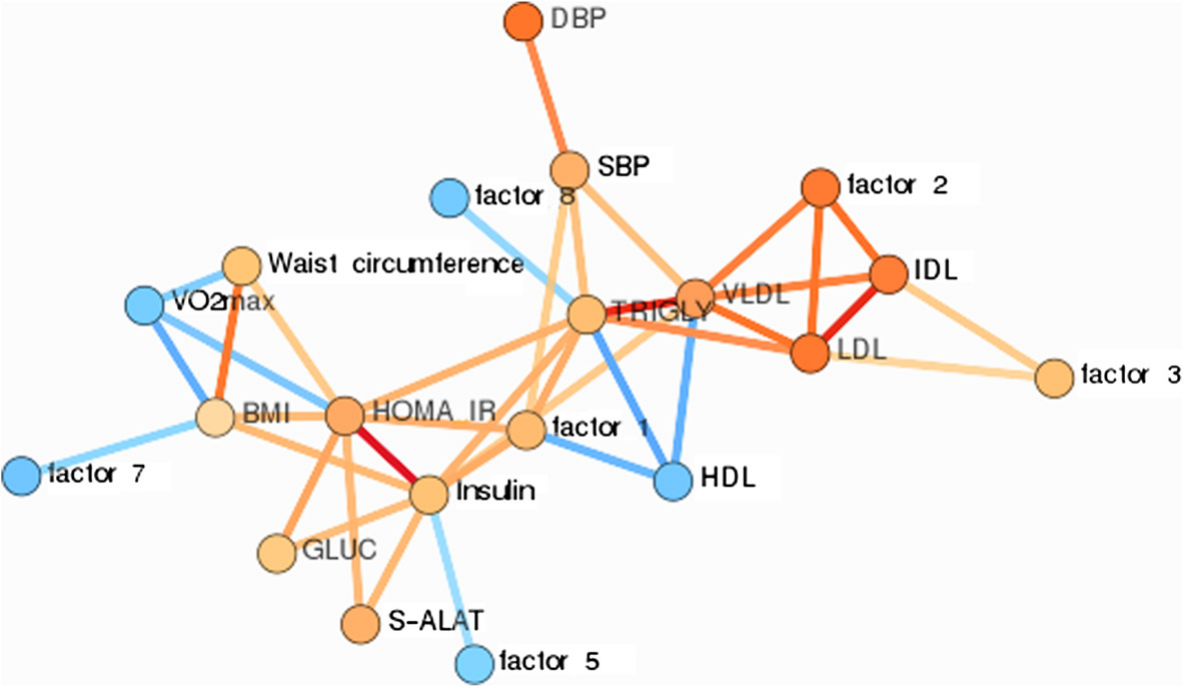
**A pruned visualization of the correlation network from un-adjusted Spearman correlation analysis.** Each variable was converted to a surrogate linear predictor before computations. The color of the edges indicate the association magnitude as shown in the legend. The vertices are colored as red and blue if all edges of the vertex are positive or negative correlations, respectively. In the cases where the vertex has both negative and positive correlations with its neighbor, the vertex is colored orange. Abbreviations: SBP = systolic blood pressure; DBP = diastolic blood pressure; VLDL = triacylglycerol and cholesterol in very-low density lipoprotein particles; LDL = triacylglycerol and cholesterol in low density lipoprotein particles; IDL = triacylglycerol and cholesterol in intermediate-density lipoprotein particles; Factor 1 (leucine, isoleucine, valine, tyrosine, phenylalanine, orosomucoid); Factor 2 (total fatty acids, omega-6 fatty acids, omega7 and 9 fatty acids, linoleic acid, mono-unsaturated fatty acids, total phosphoglycerides, total phosphocholines); Factor 3 (docosahexaenoic acid, polyunsaturated fatty acids, omega-3 fatty acids); Factor 5 (glutamine, glycine, pyruvate); Factor 6 (acetate, histidine); Factor 7 (creatinine, citrate); Factor 8 (urea).

Finally, we performed a network analysis to explore relationships between metabolite factors and clinical risk factors (Figure 2). When examining all subjects together, factor 1 was associated with HOMA-IR, insulin, triacylglycerol, SBP, VLDL, BMI, waist circumference, S-ALAT and inversely with HDL (p < 0.01 for all). Factor 2 and factor 3 were associated with IDL, LDL and VLDL, while factor 2 was also associated with SBP and triacylglycerol (p < 0.01 for all). Factor 7 was inversely associated with BMI and waist circumference, while factors 5 and 8 were inversely associated with insulin and triacylglycerol, respectively (p < 0.01 for all). All correlation coefficients and p-values are given in Additional file 3: Table S3.

## Discussion

In this study, we aimed to identify metabolite profiles that distinguish physically inactive individuals who are metabolically healthy from those who have MetS independent of fat mass. We found that two metabolite factors composed of 1) branched-chain amino acids (BCAAs), aromatic amino acids (AAAs), orosomucoid and 2) several species of fatty acids and phospholipids were associated with MetS. Factor 1 was associated with all clinical risk factors suggesting that serum amino acids and orosomucoid may be relevant biomarkers of obesity associated cardiometabolic disorders.

The risk for developing metabolic disorders is proportional to the degree of obesity [22]. However, a subset of obese individuals seems to be protected from metabolic disorders, despite having excess fat mass [23]. Consequently, factors or mechanisms that explain the development of MetS remain poorly understood, and are under intense investigation since their understanding may help design novel therapeutic strategies. The large variation in susceptibility and age of onset in individuals with a similar risk profile, suggests both genetic and environmental factors contribute to development of metabolic disorders [24]. Emerging evidence suggests several potential mechanisms contributing to MetS including dysregulation of the hypothalamic-pituitary-adrenal (HPA) axis due to chronic stress [25], dysregulation of the adipose tissue and increased cytokine production [26], the consequent systemic low-grade inflammatory state [27] and increased cellular oxidative stress [28]. Recent studies suggest that all of these mechanisms may be acting at different time during gestation, permanently reprogramming the structure and physiology of the offspring toward the development of metabolic disorders gradually progressing into a constellation of metabolic disorders in adulthood [29].

Recent studies have found that and elevated serum BCAAs [30,31] are associated with metabolic disorders independent of body weight. Our results are in agreement with the above-cited studies by showing that two factors (factor 1: BCAA, AAA and orosomucoid and factor 2: several species of fatty acids and phospholipids) were significantly different between MHO and MetS, independent of age, waist circumference and BMI. The two factors increased the risk for MetS with similar magnitude (OR 2.90 vs. 2.67, Table 3) in the present study. However, the network analysis (Figure 2) showed that factor 2 correlated only with systolic blood pressure and serum lipids and lipoproteins, whereas factor 1 was associated with all risk determinants, with most pronounced associations with triacylglycerol, insulin, HOMA-IR, S-ALAT and HDL. These findings indicate that elevated serum BCAAs and AAAs are not only associated with insulin resistance as shown in prior studies [32-34] but that they are also closely related with lipid metabolism. This notion is in agreement with a recent animal study, which showed that oral administration of BCAAs increased lipogenic gene expression and synthesis of triacylglycerol in the liver [35]. Furthermore, the association of Factor 1 with S-ALAT also suggest potential relationship for BCAAs and AAAs with fatty liver [36], which is an important predictor of several components of metabolic syndrome [37]. However, whether the amino acids are causally implicated with increased serum lipids and fatty liver in humans remains uncertain and warrants further investigation.

**Table 3:** Logistic regression model for individual factors

**Factor**	**OR**	**95% CI**	**p-value**
1) Amino acids and glycoproteins	2.90	1.40 - 6.03	0.004
2) Fatty acids and phospholipids	2.67	1.34 - 5.32	0.005
3) PUFA	1.14	0.62 - 2.09	0.667
4) Ketone bodies	0.77	0.43 - 1.39	0.385
5) Gluconeogenic intermediates	1.00	0.57 - 1.75	0.991
6) Miscellaneous	0.80	0.46 - 1.40	0.437
7) Miscellaneous	0.93	0.53 - 1.63	0.804
8) Miscellaneous	0.70	0.39 - 1.25	0.227

Component levels as predictors of MetS adjusted for age, BMI and waist circumference.

Factor 1(leucine, isoleucine, valine, tyrosine, phenylalanine, orosomucoid); Factor 2 (total fatty acids, omega-6 fatty acids, omega7 and 9 fatty acids, linoleic acid, mono-unsaturated fatty acids, total phosphoglycerides, total phosphocholines); Factor 3 (docosahexaenoic acid, polyunsaturated fatty acids, omega-3 fatty acids); Factor 4 (acetoacetate, 3-hydroxybutyrate); Factor 5 (glutamine, glycine, pyruvate); Factor 6 (acetate, histidine); Factor 7 (creatinine, citrate); Factor 8 (urea). OR = odds ratio.

It is unclear why factor 1 and its various components are present in higher concentrations in individuals with MetS. The acute phase protein (orosomucoid) is induced by infection and inflammation, and elevated plasma levels have been found in patients with type 2 diabetes [38]. Although the role of orosomucoid in the circulation is not well understood, it has been suggested to modulate immune responses to protect adipose tissue from inflammation and metabolic dysfunction [39]. As glucose [40] and lipid metabolites [41] are potential stimulants for inflammatory pathways in both adipocytes and macrophages, it is possible that the higher serum orosomucoid concentration in MetS group is attributed to higher level of these metabolic risk factors.

Recent omics-studies have shown that increased long-term leisure-time physical activity is associated with low BCAA concentration [42], and high muscle BCAA degradation [43]. Furthermore, it has been shown that high intrinsic aerobic endurance capacity is associated with higher resting metabolic rate, improved signature of muscle BCAA degradation and lower risk for MetS [44]. In the current study, all participants were physically inactive, had similar aerobic fitness and resting metabolic rate. Furthermore, although VO2max was inversely associated with triacylglycerol, HOMA-IR, BMI and waist circumference, no associations with metabolite factors were found. Dietary patterns are also significant determinants of the circulating levels of metabolites during fast. In the current study, no differences were found in dietary energy or energy yielding nutrient intakes. Thus, it is unlikely that the higher level of factor 1 and it various components can be explained by low level of physical activity or poor cardiorespiratory fitness or dietary intake. However, it could be that some biological/genetic difference in BCAA catabolism reflects the circulating BCAA concentrations in the present study. Since tissue biopsies were not obtained in the present study, we were unable to measure genetic variations of the genes encoding BCAA catabolic enzymes in skeletal muscle or adipose tissues, and thus we cannot verify whether the differences in amino acid concentrations between the groups were attributable to subtle alterations in expression of the genes in BCAA catabolic pathway.

Although, the biological basis and clinical feasibility of MetS are still debatable [45], in the present study serum metabolite profiles were significantly different between the MHO and MetS. Consequently, our results tend to suggest that serum BCAA, AAA, orosomucoid and fatty acids may be relevant determinants of metabolic health independent of fat mass and physical activity. However, our results must be interpreted in the light of the study limitations. First, the cross-sectional study design does not show temporal relationship between the studied clinical risk factors and serum metabolites and therefore causal relationship cannot be deduced but rather serves to generate hypotheses. It is also important to note that in general population, all individuals with the MetS do not necessarily have all the features described in the present study. Moreover, this study is also limited by the relatively small number of participants and the fact that the study participants only consist of Finnish women. Finally, although HOMA-IR is a widely accepted measure of insulin resistance, other methods such as hyperinsulinemic-euglycemic clamp technique is considered more robust method to measure insulin resistance [46].

In summary, our results showed that two metabolite factors were associated with MetS independent of BMI, fat mass, waist circumference and physical activity/fitness. Especially factor comprising BCAA, AAA and orosomucoid seems auspicious biomarker determining metabolic health as it was associated with all clinical risk factors. Further research is needed to determine the public health and clinical significance of these results in terms of screening to identify those at greatest cardio-metabolic risk for whom appropriate intervention strategies should be developed.

## Competing interests

The authors have nothing to declare.

## Authors’ contributions

The authors contributed to this manuscript in a following manner: PW participated in data collection, analyzed data and wrote the manuscript. SP participated in writing and editing the manuscript. RA carried out the clustering and network analyses and participated in writing the manuscript. EM coordinated the study, participated in measurements and carried out biochemical analyses. SMC carried out biochemical analyses and participated in measurements. LX, JS and UMK participated in writing and editing manuscript. MA participated in study design and writing and editing the manuscript, SC designed the study and participated in data analysis and writing and editing the manuscript. All authors were involved in writing the paper and had final approval of the submitted and published versions.

## Supplementary Material

Additional file 1: Table S1 Serum lipid constituents and low molecular-weight metabolites of study population stratified by MHO and MetS categories. Click here for file

Additional file 2: Table S2 Serum lipoprotein subclasses in study population stratified by MHO and MetS categories. Click here for file

Additional file 3: Table S3 Correlations between metabolite factors and clinical risk factors. Click here for file
