# Supplementary material for: Serum metabolic profiles in overweight and obese women with and without metabolic syndrome
Source: Diabetol Metab Syndr. 2014 Mar 20;6:40. doi: 10.1186/1758-5996-6-40 (PMC3998195; doi:10.1186/1758-5996-6-40)
Supplement: Additional file 1: Table S1 — Serum lipid constituents and low molecular-weight metabolites of study population stratified by MHO and MetS categories. [file 1758-5996-6-40-S1.docx]

Table S1. Serum lipid constituents and low molecular-weight metabolites of study population stratified by MHO and MetS categories

|  | | MHO  n=42 | | MetS  n=36 | | |  |  | p-value adj. p-value | | | |
| --- | --- | --- | --- | --- | --- | --- | --- | --- | --- | --- | --- | --- |
| *Lipid extract constituents* |  |  |  |  |  |  |  |  |  |  |  |  |
| esterified cholesterol | 3.588 | (0.453) | | 4.096 | (0.669) | | | | 0.0002 | 0.0029 |  |  |
| free cholesterol | 1.403 | (0.190) | | 1.608 | (0.268) | | | | 0.0002 | 0.0060 |  |  |
| ω-3 fatty acids | 0.389 | (0.111) | | 0.478 | (0.158) | | | | 0.0053 | 0.0210 |  |  |
| ω-6 fatty acids | 3.540 | (0.469) | | 4.020 | (0.690) | | | | 0.0006 | 0.0008 |  |  |
| ω-7, ω-9 fatty acids | 6.669 | (0.917) | | 8.956 | (2.373) | | | | <0.0001 | <0.0001 |  |  |
| Total fatty acids | 10.59 | (1.344) | | 13.45 | (3.054) | | | | <0.0001 | <0.0001 |  |  |
| Linoleic acid | 3.086 | (0.453) | | 3.529 | (0.687) | | | | 0.0015 | 0.0008 |  |  |
| Polyunsaturated fatty acids | 1.978 | (0.384) | | 2.358 | (0.550) | | | | 0.0007 | 0.0092 |  |  |
| Docosahexaenoic acid | 0.172 | (0.051) | | 0.203 | (0.064) | | | | 0.0200 | 0.0676 |  |  |
| Monounsaturated fatty acids | 3.154 | (0.484) | | 4.371 | (1.325) | | | | <0.0001 | <0.0001 |  |  |
| Phosphoglycerides | 0.890 | (0.121) | | 1.011 | (0.212) | | | | 0.0039 | 0.0097 |  |  |
| phosphatidylcholine and other cholines | 2.198 | (0.276) | | 2.424 | (0.443) | | | | 0.0118 | 0.0242 |  |  |
| Sphingomyelins | 0.401 | (0.055) | | 0.434 | (0.059) | | | | 0.0110 | 0.0843 |  |  |
| ω-3 fatty acids/ total fatty acids (%) | 3.661 | (0.913) | | 3.548 | (0.787) | | | | 0.6542 | 0.3512 |  |  |
| ω-6 fatty acids/ total fatty acids (%) | 33.45 | (2.176) | | 30.36 | (3.645) | | | | <0.0001 | 0.0001 |  |  |
| ω-7, ω-9/total fatty acids (%) | 62.88 | (2.356) | | 66.08 | (3.626) | | | | <0.0001 | 0.0001 |  |  |
| Av.number of methylene groups in a fatty acid chain | 9.773 | (0.169) | | 9.862 | (0.162) | | | | 0.0203 | 0.0048 |  |  |
| triglycerides/phosphoglycerides | 1.221 | (0.271) | | 1.773 | (0.455) | | | | <0.0001 | <0.0001 |  |  |
| Av. number of methylene groups per double bond | 7.945 | (0.563) | | 8.371 | (0.543) | | | | 0.0002 | 0.0011 |  |  |
| Av. number of double bonds in a fatty acid chain | 1.235 | (0.067) | | 1.182 | (0.064) | | | | 0.0001 | 0.0007 |  |  |
| bisallylic groups/double bonds (%) | 0.535 | (0.024) | | 0.517 | (0.027) | | | | 0.0031 | 0.0023 |  |  |
| bisallylic groups/total fatty acids (%) | 0.661 | (0.061) | | 0.612 | (0.062) | | | | 0.0002 | 0.0006 |  |  |
| description of average fatty acid chain length | 18.051 | (0.117) | | 17.978 | (0.130) | | | | 0.0115 | 0.0083 |  |  |
| *Low molecular-weight metabolites* |  |  | |  |  | | | |  |  |  |  |
| 3-hydroxybutyrate | 0.108 | (0.061) | | 0.099 | (0.033) | | | | 0.8159 | 0.4740 |  |  |
| Acetate | 0.080 | (0.012) | | 0.079 | (0.013) | | | | 0.7852 | 0.8003 |  |  |
| Acetoacetate | 0.060 | (0.036) | | 0.056 | (0.026) | | | | 0.8571 | 0.5093 |  |  |
| Alanine | 0.447 | (0.059) | | 0.483 | (0.048) | | | | 0.0032 | 0.0180 |  |  |
| Citrate | 0.092 | (0.015) | | 0.088 | (0.012) | | | | 0.3394 | 0.9006 |  |  |
| Creatinine | 0.066 | (0.009) | | 0.065 | (0.008) | | | | 0.6758 | 0.8449 |  |  |
| Glutamine | 0.531 | (0.081) | | 0.511 | (0.084) | | | | 0.2651 | 0.9052 |  |  |
| Glycerol | 0.082 | (0.023) | | 0.102 | (0.031) | | | | 0.0007 | 0.0160 |  |  |
| Glycine | 0.290 | (0.060) | | 0.280 | (0.055) | | | | 0.4398 | 0.1177 |  |  |
| Orosomucoid | 1.767 | (0.171) | | 1.966 | (0.249) | | | | <0.0001 | 0.0003 |  |  |
| Histidine | 0.074 | (0.009) | | 0.074 | (0.009) | | | | 0.8994 | 0.6774 |  |  |
| Isoleucine | 0.059 | (0.010) | | 0.073 | (0.015) | | | | <0.0001 | <0.0001 |  |  |
| Leucine | 0.094 | (0.014) | | 0.112 | (0.020) | | | | <0.0001 | <0.0001 |  |  |
| Valine | 0.221 | (0.032) | | 0.233 | (0.037) | | | | 0.1255 | 0.0965 |  |  |
| Phenylalanine | 0.080 | (0.011) | | 0.088 | (0.014) | | | | 0.0055 | 0.0229 |  |  |
| Pyruvate | 0.103 | (0.038) | | 0.112 | (0.042) | | | | 0.3067 | 0.6077 |  |  |
| Tyrosine | 0.060 | (0.013) | | 0.064 | (0.010) | | | | 0.0535 | 0.0249 |  |  |
| Urea | 0.053 | (0.019) | | 0.052 | (0.029) | | | | 0.7331 | 0.1757 |  |  |
| Lactate | 1.080 | (0.257) | | 1.280 | (0.373) | | | | 0.0147 | 0.0186 |  |  |

Mean (SD) concentrations of metabolites assayed in the present study. All metabolites are in mmol/l unless stated otherwise.

P-values are for 2-tailed t-tests comparing concentrations for the MHO and MetS groups with and without adjustment for age, fat mass and waist circumference.
