# Supplementary material for: Serum metabolic profiles in overweight and obese women with and without metabolic syndrome
Source: Diabetol Metab Syndr. 2014 Mar 20;6:40. doi: 10.1186/1758-5996-6-40 (PMC3998195; doi:10.1186/1758-5996-6-40)
Supplement: Additional file 2: Table S2 — Serum lipoprotein subclasses in study population stratified by MHO and MetS categories. [file 1758-5996-6-40-S2.docx]

Table S2. Serum lipoprotein subclasses in study population stratified by MHO and MetS categories

|  | MHO  n=42 | | | MetS  n=36 | | | p-value adj. p-value | |  |
| --- | --- | --- | --- | --- | --- | --- | --- | --- | --- |
| Albumin (cu) | | 0.100 | (0.006) | | 0.100 | (0.008) | 0.947 | 0.5828 | |
| XXL-VLDL-PL | | 0.002 | (0.002) | | 0.007 | (0.004) | <0.0001 | <0.0001 | |
| XXL-VLDL-L | | 0.019 | (0.014) | | 0.055 | (0.035) | <0.0001 | <0.0001 | |
| XXL-VLDL-P^*^ | | 0.566 | (0.685) | | 2.333 | (1.788) | <0.0001 | <0.0001 | |
| XXL-VLDL-TG | | 0.013 | (0.010) | | 0.038 | (0.024) | <0.0001 | <0.0001 | |
| XL-VLDL-PL | | 0.009 | (0.007) | | 0.025 | (0.014) | <0.0001 | <0.0001 | |
| XL-VLDL-TG | | 0.035 | (0.023) | | 0.086 | (0.048) | <0.0001 | <0.0001 | |
| XL-VLDL-L | | 0.055 | (0.036) | | 0.137 | (0.075) | <0.0001 | <0.0001 | |
| XL-VLDL-P^*^ | | 0.006 | (0.004) | | 0.014 | (0.008) | <0.0001 | <0.0001 | |
| L-VLDL-C | | 0.046 | (0.026) | | 0.102 | (0.053) | <0.0001 | <0.0001 | |
| L-VLDL-FC | | 0.024 | (0.013) | | 0.052 | (0.027) | <0.0001 | <0.0001 | |
| L-VLDL-PL | | 0.033 | (0.021) | | 0.080 | (0.045) | <0.0001 | <0.0001 | |
| L-VLDL-TG | | 0.111 | (0.069) | | 0.251 | (0.138) | <0.0001 | <0.0001 | |
| L-VLDL-CE | | 0.023 | (0.013) | | 0.050 | (0.026) | <0.0001 | <0.0001 | |
| L-VLDL-L | | 0.193 | (0.117) | | 0.440 | (0.238) | <0.0001 | <0.0001 | |
| L-VLDL-P^*^ | | 0.031 | (0.019) | | 0.070 | (0.038) | <0.0001 | <0.0001 | |
| M-VLDL-C | | 0.143 | (0.044) | | 0.230 | (0.080) | <0.0001 | <0.0001 | |
| M-VLDL-FC | | 0.060 | (0.021) | | 0.100 | (0.039) | <0.0001 | <0.0001 | |
| M-VLDL-PL | | 0.091 | (0.032) | | 0.153 | (0.060) | <0.0001 | <0.0001 | |
| M-VLDL-TG | | 0.217 | (0.095) | | 0.383 | (0.172) | <0.0001 | <0.0001 | |
| M-VLDL-CE | | 0.083 | (0.024) | | 0.129 | (0.042) | <0.0001 | <0.0001 | |
| M-VLDL-L | | 0.452 | (0.168) | | 0.768 | (0.309) | <0.0001 | <0.0001 | |
| M-VLDL-P^*^ | | 0.123 | (0.048) | | 0.212 | (0.089) | <0.0001 | <0.0001 | |
| S-VLDL-C | | 0.252 | (0.047) | | 0.320 | (0.064) | <0.0001 | 0.0018 | |
| S-VLDL-FC | | 0.096 | (0.019) | | 0.123 | (0.027) | <0.0001 | 0.0014 | |
| S-VLDL-PL | | 0.142 | (0.027) | | 0.184 | (0.041) | <0.0001 | 0.0008 | |
| S-VLDL-TG | | 0.223 | (0.061) | | 0.329 | (0.109) | <0.0001 | 0.0001 | |
| S-VLDL-L | | 0.616 | (0.128) | | 0.834 | (0.203) | <0.0001 | 0.0002 | |
| S-VLDL-P^*^ | | 0.277 | (0.061) | | 0.383 | (0.102) | <0.0001 | 0.0001 | |
| XS-VLDL-PL | | 0.159 | (0.030) | | 0.192 | (0.046) | 0.0003 | 0.0277 | |
| XS-VLDL-TG | | 0.118 | (0.025) | | 0.154 | (0.045) | <0.0001 | 0.0042 | |
| XS-VLDL-L | | 0.546 | (0.095) | | 0.688 | (0.162) | <0.0001 | 0.0010 | |
| XS-VLDL-P^*^ | | 0.370 | (0.068) | | 0.474 | (0.118) | <0.0001 | 0.0011 | |
| IDL-FC | | 0.224 | (0.039) | | 0.265 | (0.054) | 0.0002 | 0.0007 | |
| IDL-PL | | 0.309 | (0.052) | | 0.378 | (0.077) | <0.0001 | 0.0087 | |
| IDL-L | | 1.190 | (0.194) | | 1.455 | (0.284) | <0.0001 | 0.0010 | |
| IDL-P^*^ | | 0.941 | (0.160) | | 1.166 | (0.243) | <0.0001 | 0.0005 | |
| L-LDL-C | | 0.963 | (0.179) | | 1.189 | (0.230) | <0.0001 | 0.0005 | |
| L-LDL-FC | | 0.267 | (0.045) | | 0.322 | (0.058) | <0.0001 | 0.0006 | |
| L-LDL-PL | | 0.343 | (0.050) | | 0.416 | (0.071) | <0.0001 | 0.0011 | |
| L-LDL-CE | | 0.697 | (0.134) | | 0.865 | (0.172) | <0.0001 | 0.0001 | |
| L-LDL-L | | 1.410 | (0.251) | | 1.743 | (0.336) | <0.0001 | 0.0006 | |
| L-LDL-P^*^ | | 1.550 | (0.273) | | 1.916 | (0.373) | <0.0001 | 0.0004 | |
| M-LDL-C | | 0.548 | (0.122) | | 0.705 | (0.152) | <0.0001 | 0.0004 | |
| M-LDL-PL | | 0.216 | (0.031) | | 0.262 | (0.041) | <0.0001 | 0.0004 | |
| M-LDL-CE | | 0.396 | (0.097) | | 0.513 | (0.119) | <0.0001 | 3.1457 | |
| M-LDL-L | | 0.809 | (0.166) | | 1.035 | (0.214) | <0.0001 | 0.0011 | |
| M-LDL-P^*^ | | 1.240 | (0.246) | | 1.581 | (0.325) | <0.0001 | 0.0002 | |
| S-LDL-C | | 0.334 | (0.081) | | 0.453 | (0.107) | <0.0001 | 0.0001 | |
| S-LDL-L | | 0.514 | (0.114) | | 0.703 | (0.165) | <0.0001 | <0.0001 | |
| S-LDL-P | | 1.429 | (0.289) | | 1.890 | (0.431) | <0.0001 | <0.0001 | |
| XL-HDL-C | | 0.234 | (0.086) | | 0.253 | (0.088) | 0.3361 | <0.0001 | |
| XL-HDL-FC | | 0.062 | (0.022) | | 0.056 | (0.019) | 0.2216 | 0.6376 | |
| XL-HDL-PL | | 0.226 | (0.087) | | 0.173 | (0.064) | 0.0022 | 0.1244 | |
| XL-HDL-TG | | 0.014 | (0.004) | | 0.018 | (0.006) | 0.0139 | 0.0008 | |
| XL-HDL-CE | | 0.168 | (0.062) | | 0.184 | (0.063) | 0.2565 | 0.0502 | |
| XL-HDL-L | | 0.476 | (0.169) | | 0.449 | (0.146) | 0.4483 | 0.4869 | |
| XL-HDL-P^*^ | | 3.872 | (1.431) | | 3.430 | (1.144) | 0.1287 | 0.2458 | |
| L-HDL-C | | 0.443 | (0.122) | | 0.328 | (0.111) | <0.0001 | 0.0001 | |
| L-HDL-FC | | 0.094 | (0.031) | | 0.062 | (0.026) | <0.0001 | <0.0001 | |
| L-HDL-PL | | 0.420 | (0.105) | | 0.323 | (0.099) | <0.0001 | 0.0001 | |
| L-HDL-CE | | 0.349 | (0.092 | | 0.265 | (0.087) | <0.0001 | 0.0002 | |
| L-HDL-L | | 0.886 | (0.234) | | 0.673 | (0.212) | <0.0001 | 0.0001 | |
| L-HDL-P^*^ | | 11.350 | (2.975) | | 8.703 | (2.719) | <0.0001 | 0.0002 | |
| M-HDL-C | | 0.506 | (0.076) | | 0.487 | (0.092) | 0.2278 | 0.9111 | |
| M-HDL-FC | | 0.095 | (0.015) | | 0.091 | (0.019) | 0.2572 | 0.6049 | |
| M-HDL-PL | | 0.439 | (0.056) | | 0.443 | (0.077) | 0.9776 | 0.6183 | |
| M-HDL-CE | | 0.413 | (0.063) | | 0.396 | (0.075) | 0.1926 | 0.9039 | |
| M-HDL-L | | 0.975 | (0.132) | | 0.971 | (0.170) | 0.7630 | 0.6462 | |
| M-HDL-P^*^ | | 17.888 | (2.379) | | 18.100 | (3.198) | 0.8970 | 0.4554 | |
| S-HDL-TG | | 0.033 | (0.009) | | 0.044 | (0.015) | <0.0001 | 0.0023 | |
| S-HDL-L | | 1.147 | (0.092) | | 1.175 | (0.117) | 0.2712 | 0.3337 | |
| S-HDL-P^*^ | | 43.46 | (3.091) | | 44.61 | (3.855) | 0.1575 | 0.1285 | |
| VLDL-TG | | 0.694 | (0.254) | | 1.193 | (0.493) | <0.0001 | <0.0001 | |
| IDL-TG | | 0.122 | (0.028) | | 0.151 | (0.044) | 0.0007 | 0.0214 | |
| IDL-C | | 0.763 | (0.119) | | 0.910 | (0.160) | <0.0001 | 0.0009 | |
| LDL-C | | 1.841 | (0.381) | | 2.353 | (0.489) | <0.0001 | 0.0002 | |
| VLDL-D | | 36.193 | (0.970) | | 37.222 | (0.937) | <0.0001 | <0.0001 | |
| LDL-D | | 23.593 | (0.147) | | 23.494 | (0.125) | 0.0018 | 0.0059 | |
| HDL-D | | 9.994 | (0.166 | | 9.874 | (0.150) | 0.0012 | 0.0020 | |
| VLDL-TG-eFR | | 0.599 | (0.213) | | 1.061 | (0.474) | <0.0001 | <0.0001 | |
| IDL-C-eFR | | 0.206 | (0.051) | | 0.329 | (0.120) | <0.0001 | <0.0001 | |
| LDL-C-eFR | | 3.050 | (0.461) | | 3.515 | (0.569) | 0.0001 | 0.0045 | |
| HDL2-C | | 1.090 | (0.223) | | 0.934 | (0.216) | 0.0006 | 0.0023 | |
| HDL3-C | | 0.542 | (0.025) | | 0.568 | (0.054) | 0.0088 | 0.0134 | |

Mean (SD) concentrations of metabolites assayed in the present study. All metabolites are in mmol/l unless stated otherwise. P-values are for 2-tailed t-tests comparing concentrations for the MHO and MetS groups with and without adjustment for age, fat mass and waist circumference. Abbreviations: VLDL: very-low-density lipoprotein; IDL: intermediate –density lipoprotein; LDL: low-density lipoprotein; HDL: high-density lipoprotein; XXL: extremely large; XL: very large; L: large; M: medium; S: small; XS: very small; L: total protein lipids; PL: phospholipids; C: cholesterol; CE: cholesterol esters; FC: free cholesterol; TG: triglycerides; D: mean diameter; eFR=values estimated with the extended friedewald method; cu: standardized concentration units; *=nmol/l. Differences with p<0.0005 are marked in bold.
