# Supplementary material for: Serum metabolic profiles in overweight and obese women with and without metabolic syndrome
Source: Diabetol Metab Syndr. 2014 Mar 20;6:40. doi: 10.1186/1758-5996-6-40 (PMC3998195; doi:10.1186/1758-5996-6-40)
Supplement: Additional file 3: Table S3 — Correlations between metabolite factors and clinical risk factors. [file 1758-5996-6-40-S3.docx]

Table S3. Correlations between metabolite factors and clinical risk factors

| HEAD | TAIL | Correlation coefficient | P-value |
| --- | --- | --- | --- |
| BMI | VO2max | -0.493 | <0.0001 |
| BMI | Insulin | 0.415 | <0.001 |
| BMI | HOMA-IR | 0.398 | <0.001 |
| factor 1 | TRIGLY | 0.461 | <0.0001 |
| factor 1 | Insulin | 0.457 | <0.0001 |
| factor 1 | HDL | -0.455 | <0.0001 |
| factor 1 | HOMA-IR | 0.448 | <0.0001 |
| factor 1 | VLDL | 0.412 | <0.0001 |
| factor 1 | BMI | 0.382 | <0.0001 |
| factor 1 | S-ALAT | 0.360 | <0.0001 |
| factor 1 | SBP | 0.300 | <0.0001 |
| factor 2 | IDL | 0.726 | <0.0001 |
| factor 2 | LDL | 0.719 | <0.0001 |
| factor 2 | VLDL | 0.671 | <0.0001 |
| actor 2 | TRIGLY | 0.533 | <0.0001 |
| factor 2 | SBP | 0.290 | <0.05 |
| factor 3 | IDL | 0.311 | <0.01 |
| factor 3 | LDL | 0.240 | <0.05 |
| factor 5 | Insulin | -0.233 | <0.05 |
| factor 7 | BMI | -0.297 | <0.05 |
| factor 8 | TRIGLY | -0.277 | <0.05 |
| GLUC | HOMA-IR | 0.487 | <0.0001 |
| GLUC | Insulin | 0.383 | <0.001 |
| GLUC | VO2max | -0.294 | <0.05 |
| GLUC | BMI | 0.266 | <0.05 |
| GLUC | TRIGLY | 0.259 | <0.05 |
| HDL | TRIGLY | -0.519 | <0.0001 |
| HDL | VLDL | -0.482 | <0.0001 |
| HDL | Insulin | -0.238 | <0.05 |
| HDL | VO2max | 0.233 | <0.05 |
| HDL | BMI | -0.225 | <0.05 |
| S-ALAT | Insulin | 0.413 | <0.001 |
| S-ALAT | HOMA-IR | 0.401 | <0.001 |
| S-ALAT | BMI | 0.316 | <0.01 |
| S-ALAT | Waist circumference | 0.300 | <0.01 |
| S-ALAT | TRIGLY | 0.262 | <0.05 |
| SBP | DBP | 0.615 | <0.0001 |
| SBP | TRIGLY | 0.366 | <0.001 |
| SBP | VLDL | 0.365 | <0.001 |
| SBP | LDL | 0.355 | <0.01 |
| SBP | IDL | 0.290 | <0.01 |
| SBP | GLUC | 0.285 | <0.05 |
| SBP | BMI | 0.248 | <0.05 |
| TRIGLY | LDL | 0.597 | <0.0001 |
| TRIGLY | IDL | 0.522 | <0.0001 |
| TRIGLY | Insulin | 0.437 | <0.0001 |
| TRIGLY | HOMA-IR | 0.434 | <0.0001 |
| TRIGLY | VO2max | -0.232 | <0.05 |
| Waist circumference | BMI | 0.707 | <0.0001 |
| Waist circumference | VO2max | -0.356 | <0.01 |
| Waist circumference | Insulin | 0.323 | <0.01 |
| Waist circumference | HOMA-IR | 0.318 | <0.01 |
| VLDL | LDL | 0.716 | <0.0001 |
| VLDL | IDL | 0.662 | <0.0001 |
| VLDL | Insulin | 0.317 | <0.01 |
| VLDL | HOMA-IR | 0.305 | <0.01 |
| VO2max | HOMA-IR | -0.377 | <0.001 |
| VO2max | Insulin | -0.331 | <0.01 |
